# Occupational Exposure to Mycotoxins in Swine Production: Environmental and Biological Monitoring Approaches

**DOI:** 10.3390/toxins11020078

**Published:** 2019-02-01

**Authors:** Susana Viegas, Ricardo Assunção, Carla Martins, Carla Nunes, Bernd Osteresch, Magdalena Twarużek, Robert Kosicki, Jan Grajewski, Edna Ribeiro, Carla Viegas

**Affiliations:** 1 H&TRC—Health & Technology Research Center, ESTeSL—Escola Superior de Tecnologia da Saúde, Instituto Politécnico de Lisboa, 1990-096 Lisbon, Portugal; edna.ribeiro@estesl.ipl.pt (E.R.); carla.viegas@estesl.ipl.pt (C.V.); 2 Centro de Investigação em Saúde Pública, Escola Nacional de Saúde Pública, Universidade NOVA de Lisboa, 1600-560 Lisbon, Portugal; carla.martins@insa.min-saude.pt (C.M.); CNunes@ensp.unl.pt (C.N.); 3 Food and Nutrition Department, National Institute of Health Doutor Ricardo Jorge, I.P. (INSA), Av. Padre Cruz, 1649-016 Lisbon, Portugal; ricardo.assuncao@insa.min-saude.pt; 4 Centre for Environmental and Marine Studies (CESAM), University of Aveiro, Campus de Santiago, 3810-193 Aveiro, Portugal; 5 Escola Nacional de Saúde Pública, Universidade NOVA de Lisboa, 1600-560 Lisbon, Portugal; 6 Group of Prof. Humpf, Institute of Food Chemistry, Westfälische Wilhelms-Universität Münster Corrensstraße 45, 48149 Münster, Germany; osteresch@uni-muenster.de; 7 Faculty of Natural Sciences, Institute of Experimental Biology, Department of Physiology and Toxicology, Kazimierz Wielki University, 85-064 Bydgoszcz, Poland; twarmag@ukw.edu.pl (M.T.); robkos@ukw.edu.pl (R.K.); jangra@ukw.edu.pl (J.G.)

**Keywords:** mycotoxins, occupational exposure, swine production, biomonitoring, mycotoxins mixture

## Abstract

Swine production workers are exposed simultaneously to multiple contaminants. Occupational exposure to aflatoxin B_1_ (AFB_1_) in Portuguese swine production farms has already been reported. However, besides AFB_1_, data regarding fungal contamination showed that exposure to other mycotoxins could be expected in this setting. The present study aimed to characterize the occupational exposure to multiple mycotoxins of swine production workers. To provide a broad view on the burden of contamination by mycotoxins and the workers’ exposure, biological (urine) samples from workers (*n* = 25) and 38 environmental samples (air samples, *n* = 23; litter samples, *n* = 5; feed samples, *n* = 10) were collected. The mycotoxins biomarkers detected in the urine samples of the workers group were the deoxynivalenol-glucuronic acid conjugate (60%), aflatoxin M_1_ (16%), enniatin B (4%), citrinin (8%), dihydrocitrinone (12%) and ochratoxin A (80%). Results of the control group followed the same pattern, but in general with a lower number of quantifiable results (<LOQ). Besides air samples, all the other environmental samples collected presented high and diverse contamination, and deoxynivalenol (DON), like in the biomonitoring results, was the most prominent mycotoxin. The results demonstrate that the occupational environment is adding and contributing to the workers’ total exposure to mycotoxins, particularly in the case of DON. This was confirmed by the biomonitoring data and the high contamination found in feed and litter samples. Furthermore, he followed multi-biomarker approach allowed to conclude that workers and general population are exposed to several mycotoxins simultaneously. Moreover, occupational exposure is probably described as being intermittent and with very high concentrations for short durations. This should be reflected in the risk assessment process.

## 1. Introduction

The confinement buildings used for swine production are recognized for their high levels of contamination with fungi and their metabolites [1,2,3,4,5,6]. Previous studies performed in swine farms demonstrated that this environment could be considered an occupational setting with high levels of exposure to dust aerosolization [4,7,8,9], and consequently it results in the widespread presence of fungi and their metabolites, such as volatile organic compounds and mycotoxins [1,2,4,9,10,11]. Therefore, it is expected that swine production workers are exposed simultaneously to multiple contaminants, as demonstrated previously by some authors [5,8]. Besides, the swine feed contamination by mycotoxins is also a well-known and frequently reported issue in Portugal [12] and all over the world [13,14,15,16].

Occupational exposure to aflatoxin B_1_ (AFB_1_) in Portuguese swine production farms has been reported [17]. However, data regarding fungal contamination showed that exposure to other mycotoxins besides AFB_1_ could be expected in this setting. Indeed, in addition to the *Aspergillus* section *Flavi*, other fungal species recognized as mycotoxin producers were found in this occupational environment [5,10]. The most prevalent found in air (20.9%) and surface (26.6%) samples was the *Aspergillus* section *Versicolores*. However, other *Aspergillus* sections were also found, namely *Nigri*, *Circumdati* and *Fumigati* [5,10], and all of them have recognized toxigenic potential [18], besides the clinical relevance of *Fumigati* section [19].

Occupational exposure to mycotoxins is considered a complex process since it is associated with co-exposure to several mycotoxins by different exposure routes. In this context, human biomonitoring is of particular importance, characterizing the workers exposure to multiple mycotoxins and taking advantage of already available analytical methods that cover the detection and quantification of several mycotoxins and metabolites simultaneously in different biological samples [20,21,22,23]. Therefore, biomonitoring has an important role in the determination of the real human exposure to mycotoxins [17,20,22,24,25,26,27]. Biomonitoring covers not only mycotoxin intake from all dietary sources, but also exposure by other routes, such as inhalation of mycotoxins at the workplace [28]. Nowadays, the use of biomarkers has become more common, and research to discover new and more specific biomarkers has been proposed since the use of biomarkers is proven to be a successful method to assess exposure to xenobiotics. However, some challenges have to be addressed, such as the deep knowledge about the toxicokinetics and the possible metabolites for all relevant mycotoxins [29]. Other challenges include the frequent discovery of new metabolites for a specific mycotoxin and the need for understanding their possible use for biomonitoring studies, considering the measuring feasibility and the representativeness of the information regarding exposure to that mycotoxin [30]. Few studies have been performed with the use of biomarkers to study occupational exposure to mycotoxins [23,27,28].

Whether workplace-related exposure could represent a significant exposure source to mycotoxins as compared to exposure through ingestion of contaminated food constitutes a critical issue. As suggested by Reference [28], the comparison of results from workers and from non-occupationally exposed individuals (controls) should shed light on this issue contributing to the clarification of the importance of some occupational settings to multiple mycotoxins exposure in humans. The control group includes workers from administrative companies from the same locality and where the workplace environment does not have conditions to promote exposure to mycotoxins. This enables us to take into account the exposure by food intake and to have a better understanding of the role of the working environment in the total burden of mycotoxin exposure [4,24].

The present study aims to characterize the occupational exposure to multiple mycotoxins, including aflatoxin M_1_ (AFM_1_), aflatoxin B_1_ (AFB_1_), aflatoxin B_2_ (AFB_2_), aflatoxin G_1_ (AFG_1_), aflatoxin G_2_ (AFG_2_), patulin (PAT), nivalenol (NIV), deoxynivalenol (DON), deoxynivalenol-3-glucoside (DON-3-G), 15-acetyldeoxynivalenol (15-AcDON), 3-acetyldeoxynivalenol (3-AcDON), deepoxy-deoxynivalenol (DOM-1), deoxynivalenol-glucuronide (DON-GlcA), fusarenon-X (FUS-X), α-zearalanol (α-ZAL), β-zearalanol (β-ZAL), α-zearalenol (α-ZEL), β-zearalenol (β-ZEL), zearalenone (ZAN), zearalenone (ZEN), toxin T-2 (T-2), toxin HT-2 (HT-2), toxin HT-2-4-glucuronide (HT-2-4-GlcA), T-2 tetraol, T-2 triol, neosolaniol (NEO), monoacetoxyscirpenol (MAS), diacetoxyscirpenol (DAS), fumonisin B_1_ (FB_1_), fumonisin B_2_ (FB_2_), fumonisin B_3_ (FB_3_), roquefortine C (ROQ-C), griseofulvin (GRIS), ochratoxin A (OTA), ochratoxin B (OTB), ochratoxin alpha (OTα), mycophenolic acid (MPA), mevinolin (MEV), sterigmatocystin (STER), citrinin (CIT), dihydrocitrinone (DH-CIT), Enniatin B (EnB), of workers of swine production, in addition to the previously documented exposure to AFB_1_.

## 2. Results

### 2.1. Biomonitoring

#### 2.1.1. Participant Characteristics

The workers group of this study was composed of employees of five swine production farms. The volunteers of the “control group” were working in offices without expected occupational exposure to mycotoxins. The mean ages in control participants (*n* = 19) were similar to those of the workers (*n* = 25). For the control group, the median age was 40 years with a range of 32–54 years. The swine workers had a median age of 38.6 years with a range of 21–62 years (Table 1).

#### 2.1.2. Mycotoxins and Their Metabolites in Urine Samples

A summary of the biomonitoring data is presented in Table 2 and Table 3. Samples with mycotoxins biomarkers above the respective Limit of Detection (LOD) were considered positive. The mycotoxins biomarkers detected in the urine samples of workers group were DON-GlcA (60%), AFM_1_ (16%), EnB (4%), CIT (8%), DH-CIT (12%), and OTA (80%). Results for participants of the control group followed the same pattern, but in general with a lower number of positive samples (>LOD).

Here, DON 3 Glc was used as a reference that was chromatographically not separated from DON 15 GlcA, because both analytes are co-eluting in the used instrument set up. Consequently, the signal was accepted as the sum of both analytes [21,22]. As already reported in Reference [30], it is possible to separate the DON-3-GlcA, and DON-15-GlcA. However, in the instrument set-up, this would extend the liquid chromatography run up to 17 min. By doing so, the peak shapes of later eluting peaks would be worse off. It was not the aim of this study to distinguish between them, but to incorporate an early eluting polar metabolite.

Considering the values higher than LOD, DON-GlcA and OTA were the most prevalent biomarkers in the analyzed urine of the workers group, being 60% and 80% respectively. Data presented in Table 2 and Table 3 showed that glucuronidation is a metabolic pathway for DON excretion since it was detected in samples from both workers and control groups.

Most of the other mycotoxin biomarkers detected in urine samples followed a similar pattern to DON, that is, a higher proportion of positive samples (>LOD) in the workers group than in the control group (Table 4). However, the differences were not as remarkable for DON-GlcA detection. CIT and DH-CIT were also both detected in these participants, meaning that this compound is a metabolite of CIT detoxification (Table 4).

Regarding co-exposure to several mycotoxins, there are three workers that presented exposure to three mycotoxins/metabolites simultaneously: 2 workers with the combination of DON-GlcA, AFM_1_, and OTA, and 1 worker with the combination of AFM_1_, CIT, and OTA. However, the most common situation was the presence of the DON metabolite and OTA (8 workers). Regarding controls, most of the individuals showed exposure to two mycotoxins (42%) which was also the most common situation observed—the co-exposure to DON (through DON-GlcA measurement) and OTA (3 individuals). There were also 3 (21%) individuals with exposure to a mixture of 4 mycotoxins and another 3 individuals (21%) with simultaneous exposure to 3 mycotoxins.

In total, 18 (75%) workers and 15 (78%) individuals from the control group showed exposure to more than 1 mycotoxin.

### 2.2. Environmental Samples

All the collected environmental samples (air, liter, and feed) were analyzed for the presence of thirty-six mycotoxins and their metabolites (Table 5 and Table 6).

Regarding the air samples, only three samples from two different farms showed contamination by sterigmatocystin (STER) (<LOQ–1.42 ng/g). All the other air samples were found to be negative for the analyzed mycotoxins and metabolites. Regarding the litter samples, it was observed that the most prevalent mycotoxins were DON (<LOQ–76.4 ng/g) and STER (1.14–2.69 ng/g) which were detected in all litter samples and in considerably higher amounts than the other analyzed mycotoxins. Zearalenone was a mycotoxin that was also detected in 4 out of 5 farms, but in lower amounts (<LOQ–0.78 ng/g).

Concerning the feed samples, it is possible to observe that the common scenario is the co-occurrence of mycotoxins in the same sample (9–17 mycotoxins were detected in the same sample). The higher values were obtained for DON (values between 137–388 ng/g) and fumonisins, particularly FB1 (values between 6–366 ng/g). Others mycotoxins, such as ZEN, 3-AcDON, 15-AcDON, and DON-3-G, fumonisins (FB_1_, FB_2_ and FB_3_), and type A trichothecenes such as T-2 and HT-2, were also detected in almost all the feed samples.

## 3. Discussion

This study is the result of previous work related to occupational exposure to mycotoxins and the need to identify the contribution of specific occupational settings to total mycotoxins exposure. At the same time, this study and previous ones [27,31] allow us to recognize mycotoxins as real and common occupational risk factors in specific occupational settings. Indeed, as in previous reports, results showed that the occupational environment and probably specific work tasks developed by the workers implicate exposure to mycotoxins by inhalation. Although no statistical significance was obtained in some tests, results demonstrated that only workers presented quantifiable levels of DON-GlcA (a biomarker of exposure to DON), AFM_1_ (the hydroxylated metabolite of AFB_1_, EnB (also a Fusarium toxin)), DH-CIT (the main metabolite of CIT) and OTA (the most-abundant food-contaminating mycotoxin). One possible reason for the absence of statistical significance in some tests could be due to the small sample size in both groups. Additionally, the type of urine samples used for this study (spot samples) might be responsible since 24 h urine (or first-morning void) are more concentrated with mycotoxins than one spot urine sample [32]. For instance, in the case of DON, previous studies showed that there is clear evidence that urinary DON excretion varies at different times of the day, and spot samples cannot describe these differences [33,34,35].

Consequently, the results were mainly discussed in the context of their values and not their statistical significance. However, and despite the small number, results indicate that even if workers are exposed through food consumption to some of these mycotoxins, occupational exposure is adding and contributing to the total exposure. This is not difficult to understand if we consider that, besides air samples, all the other environmental samples collected presented high and diverse levels of contamination, and DON was, like in the biomonitoring results, the most prominent mycotoxin. Additionally, the almost null results regarding air samples can be explained by the fact that mycotoxins are not volatile, and for the workers, exposure by inhalation occurs when exposure to organic dust happens in specific tasks since dust functions as a mycotoxins carrier and enters respiratory systems. A previous work developed by Reference [36] identified in swine farms the predictors for dust exposure being associated with tasks involving intense animal handling, such as castrating, ear tagging, and teeth cutting, as well as activities related to feeding, floor sweeping, and removal of dry manure. If we consider the results obtained in the current study concerning the high contamination found in the litter and feed samples, it is possible to estimate that feeding, floor sweeping, and removal/change of litter will be responsible for the workers’ dust and mycotoxins exposure. Furthermore, dust particles containing mycotoxins can be deposited in the skin, leading to dermal absorption, or work surfaces contaminated with dust particles can also be touched, generating the opportunity for additional skin contact [4,37]. Consequently, this exposure route is also possible in this occupational setting since workers do not use gloves and most of the workers were using short leaves when performing their working tasks. Unfortunately, there is a lack of information on the adsorption rates from lungs and skin for mycotoxins in humans.

The results obtained regarding feed contamination (between 9–17 mycotoxins in the same sample) demonstrate that feed has a relevant role in workplace environment contamination with mycotoxins and the handling of feed is probably one of the tasks that implicates exposure. An important preventive action will be the choice of the raw materials used during feed formulation, avoiding the use of materials with high mycotoxin contaminations. Considering this aspect, it seems of interest to highlight the influence that the geographic origin of the raw material can have on the mycotoxin contamination of feed at different stages of production [38]. Previously, and similarly to our findings, DON has been reported as the more prevalent mycotoxin in the different types of raw materials used to produce feed, since it is common to find DON, for instance, in maize, wheat, soybean meal, and others [38]. This contamination has several consequences for pig health, such as increased susceptibility to infectious diseases, reactivation of chronic infection, and a decreased vaccine efficacy, with a huge economic impact on pig production [39]. Other mycotoxins present in all the feed samples analyzed, although in lower concentrations, such as ZEN, fumonisins (FB_1_, FB_2_, and FB_3_), and type A trichothecenes (T-2 and HT-2) are also commonly reported as contaminants of feed and have several health consequences for the animals [38,39]. Therefore, preventive actions taken to avoid feed contamination will result in preventing/reducing workers exposure to mycotoxins and, at the same time, guarantee better production results.

Exposure to mycotoxins mixtures was also once more revealed in this biomonitoring study. Both group results in workers and controls showed that this is a common aspect. This is understandable since, besides the presence of multiple mycotoxins in the occupational environment, this is also a common feature of food commodities. Even the most frequent combination found in biological samples from workers and controls (DON and OTA) were already reported in several foods from European countries such as beer, pasta, cereals, and cereal-based foods [26,40].

A previous paper developed by Reference [41] assessed DON and OTA interactions using two different model systems appropriate for the evaluation of intestinal or liver toxicity and an experimental design that included realistic doses of each mycotoxin. The authors found that Caco-2 and HepG2 cells were more sensitive to DON alone than to OTA. Moreover, when combined, OTA-DON showed the most toxic combinations for Caco-2 and HepG2, respectively, having both synergistic effects at all inhibition levels [41]. The same trend was found for the combination AFB_1_-DON, a mixture also observed in our study. Therefore, the results obtained in the present study, even if exposure route is mainly via inhalation, suggest that exposure to DON occurs in combination with other mycotoxins and this should be considered when performing risk assessment.

Regarding the high prevalence of OTA in the samples of both groups, previous studies developed in the Portuguese population found OTA in biologic fluids [40,42,43,44] relating to the consumption of some food commodities. Additionally, Reference [43] concluded that the estimated daily intake values in the Portuguese populations are higher than other European populations. Indeed, our results are probably explained once again by the fact that this mycotoxin is one of the most-abundant food-contaminating mycotoxins [44]. In Portugal, the bread is the major cereal-derived product consumed, and it is probably the main factor responsible for OTA exposure, also due to the contamination levels. Other products such as wine and pork also contribute to exposure but are more related to the high consumption rate of these products and not so much due to their contamination levels [44].

One aspect relevant to the analysis is the fact that in all environmental samples, including air samples, STER was detected, with a high frequency and concentration in the feed samples. STER synthesis is restricted to species in four sections in *Aspergillus* (*Ochraceorosei*, *Versicolores*, *Nidulantes,* and *Flavi*) [45]. However, most of the *Aspergillus* species from the section *Versicolores* are able to produce STER, and this was the most prevalent species on air and surface samples from the swine farms engaged in this study. Therefore, besides the feed contaminated with STER that has already been reported [45], it seems that the swine farm environment can promote this mycotoxin production by the *Versicolores* section. STER is extensively metabolized essentially by glucuronidation but the identification of the glucuronide forms in human biological samples has not been accomplished until now [29]. Further studies should be developed to determine the most suitable STER biomarkers for identifying exposure.

This study demonstrates once more the usefulness of biomonitoring tools. These tools not only allowed us to identify that the occupational environment is contributing to the swine workers’ total exposure to mycotoxins but also it revealed that exposure occurs as a mixture of mycotoxins. Furthermore, and considering that some mycotoxin mixtures could lead to additive or synergistic effects, a significant threat to human and animal health could occur. However, most studies have been carried out over less than three days and at concentrations above the legal limits available in the context of food safety. There is therefore a lack of data about chronic exposure at sub-toxic mycotoxin concentrations, closer to real food and feed consumption habits [46]. This implies also the availability of enough sensitive analytical techniques for the quantification of biomarkers of multiple co-occurring mycotoxins [47]. Likewise, and concerning occupational exposure, probably we are dealing with intermittent exposures linked with very high concentrations within a short duration of time. This exposure is in addition to the exposure occurring via food intake (chronic exposure to low amounts). Subsequently, there is a gap in the knowledge concerning the approach which should be used to accomplish a suitable risk assessment methodology. Toxicokinetics and toxicodynamics data from exposure sources other than ingestion, as well as human biomonitoring guidance values, are needed in order to anticipate the associated risk. This implies that the involved stakeholders need to extend the dialogue across different chemical sectors (food safety vs. occupational health) in order to come to more overarching and harmonized approaches [48].

Moreover, the exposure scenario found in this occupational setting can suffer variations due to climate change that will affect cereals (used for feed), agricultural practices, and the ecological niches of mycotoxigenic fungi in a particular area. In the future, mycotoxin producers in temperate climates will be replaced by better-adapted species or mutants which may produce new secondary metabolites [49,50]. Therefore, monitoring programs considering biological and environmental samples should be developed continuously to allow for a better and more detailed exposure scenario. In addition to this, adequate health surveillance programs should be applied.

## 4. Conclusions

Despite the small numbers of individuals in both groups (workers and controls), this study allowed us to recognize that the occupational environment is adding and contributing to the workers’ total exposure to mycotoxins. This was also confirmed by the high contamination found in feed and litter samples. Additionally, the multi-biomarker approach permitted us also to conclude that exposure to mycotoxins, in workers and in the general population, is characterized by being a mixture of mycotoxins, and this should be reflected in risk assessment processes.

## 5. Materials and Methods

### 5.1. Setting Characteristics

This study was conducted between June and July 2017 in five Portuguese swine locations in the Lisbon district and is part of an enlarged exploratory study aiming to characterize occupational exposure to microorganisms and mycotoxins in this setting (Instituto Politécnico de Lisboa: IPL/2016/BBIOR_ESTeSL, Date of approval: 7 December 2016). While being part of a larger study in which additional environmental characterization was carried out, this paper presents the results regarding environmental samples collected by active (air) and passive (feed and litter) methods in which mycotoxins assessment was performed. Additionally, biomonitoring was performed involving the workers who agreed to participate.

Five Portuguese swine farms were selected according to three specific criteria: Location within the Lisbon district, a high number of animals, and the number of workers. All the farms were divided into five pavilions dedicated to different phases of animal growth/age, namely pig gestation, maternity, stalls, pig fattening areas, and quarantine confinement. The five farms had been assessed in a previous study from our group [17], but no modifications in working activities or safety procedures were made until this new sampling campaign was performed in the scope of a new study. The floor in the swine maternities was covered with newspaper. Manure removal systems were present in all farm facilities, with complete removal from the building several times a day. The ventilation systems in the studied farm buildings consisted of mechanical ventilation by wall exhaust fans coupled with natural ventilation through the operation of a winch-curtain. Swine farm workers did not use respiratory protection devices during tasks performance.

Fungal burden found in the different environmental matrices from the assessed swine was already reported [5]. Besides the most prevalent (*Cladosporium* sp. and *Penicillium* sp.), other fungal species with recognized toxigenic potential were also identified, namely the *Fusarium graminearum* complex on air samples, *Fusarium culmorum* on feed samples, and *Aspergillus* section *Circumdati* on surfaces. *Aspergillus* section *Circumdati* was the most prevalent (55%) on MEA followed by *Aspergilli* (25%). Different *Aspergillus* sections were more prevalent on DG18, *Versicolores* being the most identified (50%), followed by *Usti* (20.8%).

### 5.2. Sampling

In order to provide a broad view on the burden of contamination by mycotoxins and the workers’ exposure to these toxins, biological (human biomonitoring) samples from workers (*n* = 25) and environmental (air, litter, and feed) (*n* = 38 samples) samples were collected.

#### 5.2.1. Human Biomonitoring Approach

Qualitative and quantitative determinations of mycotoxins with the objective of occupational exposure assessment at an individual level for each study participant were performed using a multi-analyte approach since it allows for a more precise and realistic exposure assessment over a broad range of different analytes [51,52]. Workers that developed tasks which implicate the handling of piglets, feed, or litter are normally inside the pavilions and were all invited to participate in this study. In the end, 25 workers (out of 26) were enrolled in this study.

A control group (not exposed) was also enrolled in the study (*n* = 19) in order to investigate mycotoxin background levels for the Portuguese population and to evaluate and easily detect putative possible differences regarding the exposure of the workers group. Therefore, the control group was composed of individuals who conducted administrative tasks in an educational institution without recognized activities known to involve or promote occupational exposure to mycotoxins [4]. Additionally, the building of the educational institution was well maintained, not showing signs of degradation that can implicate optimal conditions for fungal growth. In this study, it is assumed that both groups (workers and controls) have similar diets and consequently it was hypothesized that the main difference of exposure to mycotoxins was work activities. The same control group was used in another research project [27] since both projects were developed almost simultaneously and the workers groups are from companies located in the same region of Portugal.

This study was conducted in full accordance with the World Medical Association Declaration of Helsinki and European Commission recommendations [53,54]. Written consents from the participants involved in this study were obtained. All participants were informed about the scope and the aim of this study and signed a consent form. After data collection, all the personal data was anonymized to avoid identification of the participants. Moreover, all the data was pseudonymized in order to protect the privacy and minimize the risk in the event of unauthorized access to the participant’s data.

Additionally, during a personal interview, participants answered a questionnaire to collect personal data such as age, detailed current and previous occupational history, and tasks performed in the two previous days prior urine collection, as well as activities outside the company, e.g., agriculture or animal production. However, it only collected data needed to meet the research objectives and to obtain contextual information to enable a better analysis of the biomonitoring data. In each unit, workers collected spot urine samples (more or less 25 mL) at the end of the morning (between 11 a.m. and 1 p.m.) in a dedicated room in each swine farm facility. This schedule was the one indicated by the companies as the most suitable for samples collection.

#### 5.2.2. Environmental Sampling

Air, litter, and feed from the swine farms (identified as A, B, C, D, and E) were analyzed to assess mycotoxins contamination. The objective of considering these environmental samples was to recognize the most relevant contamination source of the occupational environment and to identify potential preventive measures that could be more adequate to reduce workers exposure to mycotoxins. In each area of the swine farms considered in the study (the pig gestation site, maternity site, stalls, the pig fattening area, and quarantine confinement) air samples were collected. In total, 23 air samples were collected. Air samples (600 L) were collected using the impinger Coriolis^®^ µ air sampler (Bertin Technologies, Montigny-le-Bretonneux, France) with a flow rate of 300 L of air per minute. Samples were collected using 10 mL sterile phosphate-buffered saline (PBS) with 0.05% Triton^tm^ X-100 and were subsequently used for the mycotoxins assay.

Five litter samples (one from each unit) were collected into sterilized bags in the maternity area, the only area off the swine farm that had litter. Ten feed samples (two from each swine farm) from different areas of the swine farms were collected into sterilized bags.

### 5.3. Analytical Methods for the Determination of Mycotoxins and Metabolites

#### 5.3.1. Urine Samples Analysis

Urine samples were stored at 4 °C after collection and during transportation to the laboratory. After aliquotation, 15 mL of these samples were kept frozen at −20 °C until analysis in the next two weeks. After the collection of all samples, dilute-and-shoot sample preparation was used that consists only of centrifugation as well as a dilution step of thawed samples in combination with a HPLC-MS/MS measurement.

In short, samples were centrifuged at 15,000× *g* for five minutes at 8 °C followed by dilution of 10 µL of the supernatant with 90 µL mobile phase at LC-starting conditions, namely a solvent mixture of acetonitrile, water, and formic acid (95+5+0.1, *v*/*v*/*v*), following the sample preparation from an earlier published approach [21]. Sample 30 µL of this solution was injected to an Infinity 1260 system (Agilent, Waldbronn, Germany) on a C18 Pyramid column (100 × 2 mm, 3 µm, Macherey-Nagel, Düren, Germany) connected to a pre-column filled with the same material (4 × 2 mm, 3 µm). Column oven temperature was set to 45 °C, and the flow rate was 600 µL/min. After chromatographic separation, the detection was carried out by a QTRAP 6500 triple quadrupole mass spectrometer (SCIEX, Santa Clara, CA, USA) run by Analyst 1.6.2 software (SCIEX, Santa Clara, CA, USA). Source parameters were as follows: Temperature was set to 500 °C, as well as curtain gas at 40, nebulizer gas at 45, and heater gas at 55 arbitrary units. Electrospray ionization was used in both polarities at −4500 V or +5500 V, respectively. Further parameters and characteristics, for example, the used gradient of the mobile phases or the Multiple Reaction Monitoring (MRM) transitions, can be found in the original publication of this method application [22]. Analytes of interest are presented in Table 3. Additionally, the presence of structurally-related compounds and important metabolites was investigated. Since spot urine samples were used to determine the workers’ exposure to mycotoxins, it was necessary to perform an adjustment in order to correct for differences in inter-individual dilution and excretion rates [27]. The determination of urinary creatinine was chosen to perform this adjustment. Creatinine was determined with a spectrophotometric method based on Jaffe reaction in automatized equipment (Dimension RXL, Siemens^®^, Munich, Germany). Results for mycotoxins urinary concentrations were expressed as µg mycotoxin/g creatinine.

#### 5.3.2. Analyses of the Environmental Samples

Aliquots from feed (0.50 g) and litter (0.25 g) were extracted with 2.0 mL of extraction solvent (acetonitrile (ACN): water (H_2_O): acetic acid (AcOH) 79:20:1) on MultiReax shaker (Heidolph, Germany) for 60 min. Raw extracts after dilution with water (1:1) and centrifugation were injected into the LC-MS/MS system. Air samples (600 L) were diluted 1:7 (*v*/*v*) with extraction solvent and water mixture (1:1) (Table 7).

Mycotoxins were detected using high-performance liquid chromatography (HPLC) Nexera (Shimadzu, Tokyo, Japan) with a mass detector API 4000 (Sciex, Foster City, CA, USA). Separation of mycotoxins was carried out on a chromatographic column Gemini NXC18 (150 × 4.6 mm, 3 μm) (Phenomenex, Torrance, CA, USA); eluent A was composed of water/acetic acid (99:1, *v*/*v*) and eluent B of methanol /acetic acid (99:1, *v*/*v*), both contained 5mM ammonium acetate; eluent flow rate: 0.75 mL/min, injection volume: 7 μL. The concentrations of mycotoxins were calculated using external calibration. The Limits of Detection (LOD) and Limits of Quantitation (LOQ) obtained for each mycotoxin with the analytical method are presented in Table 7. The LOD (signal-to-noise ratio of 3) and LOQ (signal-to-noise ratio of 10), respectively, were estimated (using the Analyst^®^ 1.6.2 software (Sciex, Foster City, CA, USA), by spiking blank feed extract before extraction at low concentrations.

### 5.4. Statistical Analysis

Statistical analysis was performed using IBM^®^ SPSS Statistics 20 software (IBM, Armonk, NY, USA). Descriptive statistics are presented as medians (IQR) and range (minimum and maximum). Assuming the research (alternative) hypothesis “there is a difference in the distribution of responses to the outcome variable among the comparison groups” (i.e., that the distribution of responses “depends” on the group), differences in the proportion of exposures between the control group and workers were evaluated through the Chi-Square Test of Independence (with continuity correction or the Fisher Exact Test—in case the conditions of the applied Chi-Square Test of Independence were not satisfied). For this, the classification of “not exposed” were considered to be the values below the LOD, and “exposed” considers the values higher than the LOD. The level of *p* ≤ 0.05 was considered statistically significant.

## Figures and Tables

**Table 1:** Participants age and years of activity.

Groups	Female	Male	Age (Median; IQR)	Years of Activity (Median; IQR)
Workers (*n* = 25)	13	12	38.6; 30.0–46.0	3.5 ± 10.1
Controls (*n* = 19)	7	12	40.0; 38.5–44.0	-

IQR = Interquartile range.

**Table 2:** Mycotoxins biomarkers detected in urine samples from workers and controls.

Groups	DON-GlcA	AFM_1_	EnB	CIT	DH-CIT	OTA
LOD (µg/L)	1.24	0.11	0.006	0.61	0.115	0.011
LOQ (µg/L)	4.14	0.38	0.020	2.00	0.383	0.036
**Workers (*n* = 25)**						
>LOQ (*n*, %)	13, 52%	4, 16%	-	1, 4%	1, 4%	1, 4%
LOD–LOQ (*n*, %)	2, 8%	-	1, 4%	1, 4%	2, 8%	19, 76%
<LOD (*n*, %)	10, 40%	21, 84%	24, 96%	23, 92%	22, 88%	5, 20%
**Controls (*n* = 19)**						
>LOQ (*n*, %)	-	-	-	1, 5%	-	-
LOD–LOQ (*n*, %)	11, 58%	1, 5%	2, 11%	10, 53%	2, 11%	13, 68%
<LOD (*n*, %)	8, 42%	18, 95%	17, 89%	8, 42%	17, 89%	6, 32%

Limit of Detection (LOD); Limit of Quantification (LOQ); Deoxynivalenol-glucuronide (DON-GlcA); Aflatoxin M_1_ (AFM_1_); Enniatin B (EnB); Citrinine (CIT); Dihydrocitrinone (DH-CIT); Ochratoxin A (OTA).

**Table 3:** Mycotoxins biomarkers levels (>LOQ) in urine samples from workers and controls (µg/L).

Groups	DON-GlcA	AFM_1_	CIT	DH-CIT	OTA
**Workers**					
Range	22.0–71.1	2.1–5.4	-	-	-
Median	32.8	4.9	-	-	-
IQR	27.2–44.5	4.5–8.1	-	-	-
Single value	-	-	5.3	0.8	0.1
**Controls (µg/L)**					
Single value			24.2		

Interquartile range (IQR); Deoxynivalenol-glucuronide (DON-GlcA); Aflatoxin M_1_ (AFM_1_); Enniatin B (EnB); Citrinine (CIT); Dihydrocitrinone (DH-CIT); Ochratoxin A (OTA).

**Table 4:** Differences in the proportion of exposures between the control group and workers group.

Mycotoxins	Groups	Total	Workers	Controls	*p* Value
DON-GlcA	Not exposed	18 (40.9%)	10 (40.0%)	8 (42.1%)	1 *
	Exposed	26 (59.1%)	15 (60.0%)	11 (57.9%)	
AFM_1_	Not exposed	39 (88.6%)	21 (84.0%)	18 (94.7%)	0.370 **
	Exposed	5 (11.4%)	4 (16.0%)	1 (5.3%)	
CIT	Not exposed	31 (70.5%)	23 (92.0%)	8 (42.1%)	0.001 *
	Exposed	13 (29.5%)	2 (8.0%)	11 (57.9%)	
DH-CIT	Not exposed	39 (88.6%)	22 (88.0%)	17 (89.5%)	1 **
	Exposed	5 (11.4%)	3 (12.0%)	2 (10.5%)	
EnB	Not exposed	41 (93.2%)	24 (96.0%)	17 (89.5%)	0.57 **
	Exposed	3 (6.8%)	1 (4.0%)	2 (10.5%)	
OTA	Not exposed	11 (25.0%)	5 (20.0%)	6 (31.6%)	0.598 *
	Exposed	33 (75%)	20 (80.0%)	13 (68.4%)	

* Chi-Square Test of Independence; ** Fisher Exact Test.

**Table 5:** Mycotoxins present in environmental samples.

Farms	Environmental Samples	Mycotoxins *	Number of Mycotoxins		
			(>LOD)	LOD–LOQ	> LOQ
Farm A	Feed—Sample 1	NIV, DON-3-G, DON, ZEN, NEO, 15-AcDON, 3-Ac-DON, MAS, DAS, FB_1_, FB_2_, FB_3_, GRI, T-2, HT-2, MPA, STER	17	2	15
	Feed—Sample 2	DON, ZEN, 15-AcDON, 3-AcDON, FB_1_, FB_2_, FB_3_, T-2, HT-2, MPA, MEV	11	0	11
	Air				
	Litter	DON, ZEN, FB_1_, STER	4	0	4
Farm B	Feed—Sample 1	DON-3-G, DON, ZEN, 15-AcDON, FB_1_, FB_2_, FB_3_, T-2, HT-2, OTA, MPA, MEV	12	1	11
	Feed – Sample 2	DON-3-G, DON, ZEN, 15-AcDON, 3-AcDON, MAS, FB_1_, FB_2_, FB_3_, T-2, HT-2, MPA	12	3	9
	Air				
	Litter	DON, ZEN, GRI, STER	4	0	4
Farm C	Feed—Sample 1	DON, ZEN, NEO, 15-AcDON, FB_1_, FB_2_, FB_3_, GRI, T-2, HT-2, MPA	11	0	11
	Feed—Sample 2	DON-3-G, DON, ZEN, 15-AcDON, FB_1_, FB_2_, FB_3_, T-2,HT-2, MPA, MEV	11	3	8
	Air				
	Litter	DON, ZEN, DOM-1, STER	4	0	4
Farm D	Feed—Sample 1	DON-3-G, DON, ZEN, 15-AcDON, FB_1_, FB_2_, FB_3_, T-2, HT-2, MPA, MEV	11	1	10
	Feed—Sample 2	DON, ZEN, 15-AcDON, FB_1_, FB_2_, FB_3_, T-2, HT-2, MPA	9	1	8
	Air				
	Litter	DON, ZEN, FB_1_, GRI, STER	5	0	5
Farm E	Feed—Sample 1	DON-3-G, DON, ZEN, NEO, 15-AcDON, FB_1_, FB_2_, FB_3_, T-2, HT-2, MPA	11	1	10
	Feed—Sample 2	DON, ZEN, 15-AcDON, FB_1_, FB_2_, T-2, HT-2, MPA, STER	9	1	8
	Air				
	Litter	DON, GRI, STER, MPA	4	0	4

* Mycotoxins with values >LOD; nivalenol (NIV), deoxynivalenol (DON), deoxynivalenol-3-glucoside (DON-3-G), fusarenon-X (FUS-X), α-zearalanol (α-ZAL), β-zearalanol (β-ZAL), β-zearalenol (β-ZEL), α-zearalanol (α-ZEL), zearalenone (ZAN), zearalenone (ZEN), Toxin T2 (T-2), Toxin HT2 (HT-2), deepoxy-deoxynivalenol (DOM-1), neosolaniol (NEO), 15-acetyldeoxynivalenol (15-AcDON), 3-acetyldeoxynivalenol (3-AcDON), monoacetoxyscirpenol (MAS), diacetoxyscirpenol (DAS), fumonisin B_1_ (FB_1_), fumonisin B_2_ (FB_2_), fumonisin B_3_ (FB_3_), roquefortine C (ROQ-C), griseofulvin (GRI), ochratoxin A (OTA), ochratoxin B (OTB), mycophenolic acid (MPA), mevinolin (MEV), sterigmatocystin (STER).

**Table 6:** The concentration of mycotoxins quantified in the feed (ng/g).

Mycotoxins	Range of Values/Mean
NIV	<LOQ
DON-3-G	<LOQ
DON	137–388/272
ZEN	6.83–32.35/14.4
NEO	0.96–12.4/4.84
15-AcDON	6.94–35.64/14.79
3-Ac-DON	4.48–10.9/7.66
MAS	<LOQ–0.70
DAS	1.18
FB_1_	6.52–366/149
FB_2_	2.06–97.6/48.3
FB_3_	6.36–61.2/19.6
GRIS	1.59–1.88/1.74
T-2	<LOQ–24.6/3.81
HT-2	<LOQ–28.1/3.84
MPA	0.80–89.0/29.7
STER	<LOQ–0.72
MEV	0.43–0.62/0.55
OTA	0.30

Nivalenol (NIV), deoxynivalenol (DON), deoxynivalenol-3-glucoside (DON-3-G), fusarenon-X (FUS-X), α-zearalanol (α-ZAL), β-zearalanol (β-ZAL), β-zearalenol (β-ZEL), α-zearalanol (α-ZEL), zearalenone (ZAN), zearalenone (ZEN), Toxin T2 (T-2), Toxin HT2 (HT-2), deepoxy-deoxynivalenol (DOM-1), neosolaniol (NEO), 15-acetyldeoxynivalenol (15-AcDON), 3-acetyldeoxynivalenol (3-AcDON), monoacetoxyscirpenol (MAS), diacetoxyscirpenol (DAS), fumonisin B_1_ (FB_1_), fumonisin B_2_ (FB2), fumonisin B_3_ (FB_3_), roquefortine C (ROQ-C), griseofulvin (GRIS), ochratoxin A (OTA), ochratoxin B (OTB), mycophenolic acid (MPA), mevinolin (MEV), sterigmatocystin (STER).

**Table 7:** Limits of Detection (LOD) and Limits of Quantitation (LOQ) for mycotoxins analyzed by LC-MS/MS in environmental samples.

Mycotoxins	LOD (µg/Kg)	LOQ (µg/Kg)	Calibration Range	Recovery (%) ± RSD (*n* = 3)
Aflatoxin M_1_	0.06	0.20	0.1–8.1	79 ± 6
Aflatoxin B_1_	0.06	0.20	0.3–32.1	80 ± 2
Aflatoxin B_2_	0.06	0.20	0.1–8.0	101 ± 12
Aflatoxin G_1_	0.10	0.10	0.3–32.4	81 ± 2
Aflatoxin G_2_	0.12	0.40	0.1–8.0	74 ± 1
Deoxynivalenol	2.70	9.00	3.2–1060	90 ± 2
Deoxynivalenol-3-glucoside	5.41	18.00	5.5–548	85 ± 7
15-Acetyldeoxynivalenol	0.81	2.70	3.3–1100	88 ± 6
3-Acetyldeoxynivalenol	0.81	2.70	3.2–1070	90 ± 1
Deepoxydeoxynivalenol	0.36	1.20	1.7–558	92 ± 5
Nivalenol	4.50	15.00	10.7–1070	83 ± 4
Neosolaniol	0.09	0.30	2.2–740	92 ± 2
Zearalanone	0.45	1.50	3.2–107	85 ± 5
Zearalenone	0.18	0.60	0.5–151	87 ± 3
α-Zearalanol	1.98	6.60	2.0–47.4	83 ± 7
β-Zearalanol	0.93	3.10	1.0–47.2	85 ± 7
β-Zearalenol	1.44	4.80	2.0–47.2	81 ± 1
α-Zearalenol	1.02	3.40	1.0–48.6	89 ± 1
Ochratoxin A	0.06	0.20	2.0–199	103 ± 1
Ochratoxin B	0.09	0.30	1.6–164	99 ± 1
Fumonisin B_1_	0.51	1.70	8.1–811	64 ± 9
Fumonisin B_2_	0.36	1.20	8.1–809	70 ± 9
Fumonisin B_3_	0.45	1.50	2.4–235	66 ± 11
T2 toxin	0.12	0.40	3.2–319	104 ± 4
HT2 toxin	0.27	0.90	3.2–322	98 ± 1
T2 Tetraol	5.41	18.00	7.4–741	87 ± 5
T2 Triol	0.33	1.10	2.2–222	103 ± 6
Monoacetoxyscirpenol	0.12	0.40	1.9–634	93 ± 5
Diacetoxyscirpenol	0.30	1	3.2–322	97 ± 2
Roquefortine C	0.21	0.70	3.5–352	87 ± 4
Griseofulvin	0.09	0.30	2.4–239	94 ± 3
Patulin	1.05	3.50	4.1–405	93 ± 7
Fusarenon-X	4.80	16.00	6.4–319	81 ± 8
Mycophenolic acid	0.21	0.70	2.4–815	101 ± 2
Mevinolin	0.09	0.30	2.4–239	98 ± 1
Sterigmatocystin	0.20	0.60	1.0–101	100 ± 3
